# Mutations in *TBL1X* Are Associated With Central Hypothyroidism

**DOI:** 10.1210/jc.2016-2531

**Published:** 2016-09-07

**Authors:** Charlotte A. Heinen, Monique Losekoot, Yu Sun, Peter J. Watson, Louise Fairall, Sjoerd D. Joustra, Nitash Zwaveling-Soonawala, Wilma Oostdijk, Erica L. T. van den Akker, Mariëlle Alders, Gijs W. E. Santen, Rick R. van Rijn, Wouter A. Dreschler, Olga V. Surovtseva, Nienke R. Biermasz, Raoul C. Hennekam, Jan M. Wit, John W. R. Schwabe, Anita Boelen, Eric Fliers, A. S. Paul van Trotsenburg

**Affiliations:** Department of Endocrinology and Metabolism (C.A.H., O.V.S., A.B., E.F.), Clinical Genetics (M.A.), and Clinical and Experimental Audiology (W.A.D.), Academic Medical Centre, University of Amsterdam, 1100 DD Amsterdam, The Netherlands; Departments of Paediatric Endocrinology (C.A.H., N.Z.-S., A.S.P.v.T.), Radiology (R.R.v.R.), and Paediatrics (R.C.H.), Emma Children's Hospital, Academic Medical Centre, University of Amsterdam, 1100 DD Amsterdam, The Netherlands; Departments of Clinical Genetics (M.L., Y.S., G.W.E.S.), Paediatrics (S.D.J., W.O., J.M.W.), and Endocrinology and Metabolism (S.D.J., N.R.B.), Leiden University Medical Centre, 2300 RC Leiden, The Netherlands; Henry Wellcome Laboratories of Structural Biology (P.J.W., L.F., J.W.R.S.), Department of Molecular and Cell Biology, University of Leicester, Leicester LE1 7RH, United Kingdom; and Department of Paediatric Endocrinology (E.L.T.v.d.A.), Erasmus Medical Centre, 3000 CB Rotterdam, The Netherlands

## Abstract

**Context:**

Isolated congenital central hypothyroidism (CeH) can result from mutations in *TRHR*, *TSHB*, and *IGSF1*, but its etiology often remains unexplained. We identified a missense mutation in the transducin β-like protein 1, X-linked (*TBL1X*) gene in three relatives diagnosed with isolated CeH. TBL1X is part of the thyroid hormone receptor-corepressor complex.

**Objective:**

The objectives of the study were the identification of *TBL1X* mutations in patients with unexplained isolated CeH, Sanger sequencing of relatives of affected individuals, and clinical and biochemical characterization; in vitro investigation of functional consequences of mutations; and mRNA expression in, and immunostaining of, human hypothalami and pituitary glands.

**Design:**

This was an observational study.

**Setting:**

The study was conducted at university medical centers.

**Patients:**

Nineteen individuals with and seven without a mutation participated in the study.

**Main Outcome Measures:**

Outcome measures included sequencing results, clinical and biochemical characteristics of mutation carriers, and results of in vitro functional and expression studies.

**Results:**

Sanger sequencing yielded five additional mutations. All patients (n = 8; six males) were previously diagnosed with CeH (free T_4_ [FT4] concentration below the reference interval, normal thyrotropin). Eleven relatives (two males) also carried mutations. One female had CeH, whereas 10 others had low-normal FT4 concentrations. As a group, adult mutation carriers had 20%–25% lower FT4 concentrations than controls. Twelve of 19 evaluated carriers had hearing loss. Mutations are located in the highly conserved WD40-repeat domain of the protein, influencing its expression and thermal stability. TBL1X mRNA and protein are expressed in the human hypothalamus and pituitary.

**Conclusions:**

*TBL1X* mutations are associated with CeH and hearing loss. FT4 concentrations in mutation carriers vary from low-normal to values compatible with CeH.

## Abstract (precis)

By using DNA-analysis, clinical and biochemical phenotyping, and in vitro functional and expression studies, we show that TBL1X mutations are associated with central hypothyroidism and hearing loss.

Central hypothyroidism (CeH) is characterized by suboptimal thyroid hormone (TH) secretion due to insufficient stimulation by TSH of an otherwise normal thyroid gland. CeH may be caused by congenital or acquired disorders of the pituitary gland or hypothalamus (1). The diagnosis is based on a plasma free T_4_ (FT4) concentration below the reference interval in combination with an inappropriately normal TSH. Congenital CeH has an estimated incidence of 1 in 18 000 and is isolated in 25% of cases (2). Until now, three genetic causes of isolated CeH have been discovered: mutations in *TRHR, TSHB*, and *IGSF1* (3–5). Yet the etiology of most cases of isolated disease has remained unexplained.

In our ongoing search for other genetic causes, we studied three patients from one family with isolated CeH who tested negative for mutations in *TRHR*, *TSHB*, and *IGSF1*. In these patients, we identified a missense mutation in the gene encoding transducin β-like protein 1, X-linked (*TBL1X*). Sanger sequencing of *TBL1X* in 50 other patients with unexplained isolated CeH yielded five other missense mutations in five families.

TBL1X is an essential subunit of the nuclear receptor corepressor (NCoR)-silencing mediator for retinoid and thyroid hormone receptors (SMRT) complex, the major TH receptor (TR) corepressor (CoR) involved in T_3_-regulated gene expression. Disruption of NCoR in mice was found to result in decreased TH synthesis while possibly increasing peripheral sensitivity to TH (6). In humans, *TBL1X* deletions have been associated with hearing loss (7, 8) but not with CeH.

Here we report the phenotype of the probands and relatives with a mutation in *TBL1X* and the results of structural and functional studies of the mutated TBL1X protein.

## Materials and Methods

### Acquisition of patients

**Figure 1.**
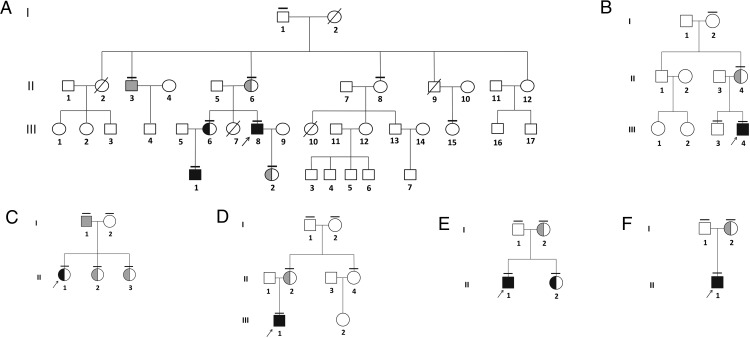
Pedigrees of the six families in which *TBL1X* mutations were found. Probands are indicated by an arrow, and small horizontal lines indicate that mutation analysis was performed. Black and gray filled symbols represent mutation-carrying individuals with CeH and euthyroidism, respectively. Pedigree of family A (A), family B (B), family C (C), family D (D), family E (E), and family F (F) is shown.

In ongoing studies on X-linked CeH, we performed X-exome sequencing in three patients with CeH and two relatives from one family (family A, Figure 1A). The 25-year-old proband (A.III.8) and his sister's 1.5-year-old son (A.IV.1) were diagnosed with CeH after detection by the Dutch T_4_-based neonatal congenital hypothyroidism (CH) screening (2). They were treated with levothyroxine (LT4) from the ages of 6 months and 16 days, respectively. The proband's sister (A.III.6) was diagnosed with CeH when she was 27 years old and was subsequently treated with LT4. An overview of the X-exome sequencing results is summarized in Supplemental Table 1. After identification of a potentially pathogenic *TBL1X* variant in these patients, Sanger sequencing was performed on DNA samples from 50 unrelated patients with idiopathic CeH, resulting in the discovery of five other mutations in five patients. Through family studies, 11 other individuals with a mutation were detected. Written informed consent was obtained in all cases.

### Phenotyping

All individuals with a mutation were phenotyped in detail, including assessment of growth and development, biochemical evaluation of endocrine axes (see Supplemental Material), brain magnetic resonance imaging, thyroid and testicular ultrasound, and pure tone audiometry (PTA) or otoacoustic emission testing. Because mice with a loss of hepatic TBL1X show hepatic hypertriglyceridemia and steatosis (9), plasma liver enzymes and lipids were measured. PTA was performed in a soundproof booth, using a manual audiometer (Madsen Electronics) with TDH-39 headphones, calibrated according to International Organization for Standardization-389-1, with adequate masking (10). To compare hearing thresholds between groups, we used the air conduction thresholds, corrected for gender and age, according to the International Organization for Standardization 1999 (11). Seven relatives without a mutation were invited for evaluation of the hypothalamus-pituitary-thyroid (HPT) axis, thyroid ultrasound, and PTA to serve as controls. A two-way, repeated-measures ANOVA analysis was used to compare the corrected thresholds of individuals with a mutation with those of relatives without a mutation. A value of *P* < .05 was considered to be significant. SPSS version 22 for Windows (SPSS, Inc) was used. The Medical Ethics Committee of the Academic Medical Centre (Amsterdam, The Netherlands) approved the study protocol (NL47462.018.13).

### Genetic analyses

Genomic DNA isolation and X-exome enrichment were performed as described previously (5). The KAPA library preparation kit (Illumina) was used to prepare the DNA for X-exome sequencing on the Illumina MiSeq-generating, 250-bp paired-end reads. Read mapping, variant calling, annotation, and filtering strategy were essentially as described earlier (12). Candidate variants were confirmed by Sanger sequencing, and Sanger sequencing of the complete coding region of *TBL1X* in unrelated individuals with CeH was performed using standard procedures (conditions and primer sequences are available upon request). All available first- and second-degree relatives of the probands were invited for molecular analysis. X-chromosome inactivation analysis was performed in females with a mutation as described previously (13).

Genes known or expected to cause isolated CeH (*TSHB*, *TRHR*, *IGSF1*) were sequenced using Sanger sequencing in the probands of all families. Whole-exome sequencing (WES) and variant calling were performed by Beijing Genomics Institute using the Complete Genetics platform in all individuals with a mutation and CeH from families A–D to evaluate the presence of potentially pathogenic variants in other genes. Rare variants were identified by focusing on protein-altering and splice-site changing mutations that were present at a frequency of less than 1% in the general population (based on dbSNP database [dbSNP build 141 GRCh37.p13], ESP6500 [http://evs.gs.washington.edu/EVS/], 1000 Genomes project [1000 Genomes phase 3 release version 5.20130502], GoNL [http://www.nlgenome.nl/], and more than 900 in-house reference samples). Missense mutations that were not likely to be pathogenic based on in silico prediction (Sorting Intolerant From Tolerant score >0.1, and Polymorphism Phenotyping prediction <0.90) were discarded. All variants were additionally checked against a list of genes that are known or presumed to be involved in HPT axis functioning based on their position in biological pathways, expression, or animal models (Supplemental Table 2C). In family A, displaying vertical transmission, all variants present in each of the three affected members were checked against variants in a gene panel consisting of genes defined to be medically relevant by the Medical Exome Project (14) and Clinical Research Exome (Agilent Technologies) or listed as disease causing in the online inheritance in man database.

### Protein structural and functional studies

To express the TBL1-histone deacetylase 3 (HDAC3)-G protein pathway suppressor 2 (GPS2)-SMRT chimera complex, full-length TBL1X and HDAC3 were cloned into the pcDNA3 vector, a chimera between GPS2 and SMRT was cloned into the pcDNA3 vector with a N-terminal 10xHis-3xFlag tag and a TEV protease cleavage site. To express TBL1X in isolation, full-length TBL1X and the TBL1X WD40 domain (amino acids 100–526) were cloned into the pcDNA3 vector with a N-terminal 10xHis-3xFlag tag and a TEV protease cleavage site. Transient transfections in mammalian cells and protein purifications were performed as described previously (see Supplemental Material) (15).

### mRNA expression and immunostaining

We studied eight human hypothalami and five pituitary glands obtained from the Netherlands Brain Bank, in accordance with the formal permission for the use of human brain material for research purposes. Three unfixed, frozen (−80°C) hypothalami and three pituitary glands were used for mRNA expression, and three paraformaldehyde-fixed hypothalami were used for immunocytochemistry. In addition, we used frozen hypothalamus and pituitary glands from two patients for Western blot analysis (see Supplemental Material).

## Results

### Genetic analyses

**Figure 2.**
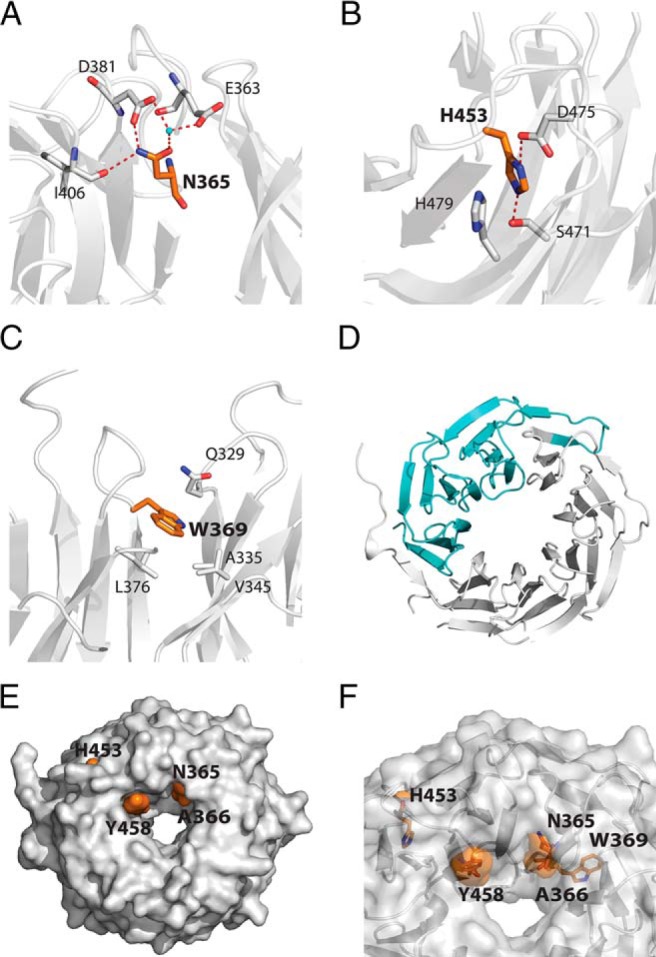
Schematic representations of the mutated amino-acids. The mutated amino-acids are shown on the crystal structure of the TBL1XR1 WD40 domain (PDB ID 4LG9). In each case the mutated residues are shown in orange, TBL1XR1 in gray, and water molecules in the crystal structure in cyan. The numbering of the amino acids is as for TBL1X. A, N365. B, H453. C, W369. D, c.1312-1G>A splice mutation with the missing amino acids in cyan (starting at asterisk). E, Surface representation of the WD40 domain to show the mutations that are on the surface. F, Transparent representation of the WD40 domain to show the buried and surface mutations.

X-exome sequencing and subsequent filtering steps yielded a single missense variant in *TBL1X* (transcript accession number NM_001139466.1) in the three patients with CeH from family A, subsequently confirmed by Sanger sequencing. Sanger sequencing of 50 other individuals with idiopathic CeH yielded mutations in five unrelated patients. None of the detected mutations were present in available databases (dbSNP, 1000 Genomes Project, Human Gene Mutation Database, National Heart, Lung, and Blood Institute Exome Sequencing Project, GoNL) or previously reported. All mutations were located in the highly conserved WD40-repeat domains of the TBL1X protein (Figure 2; National Heart, Lung, and Blood Institute reference sequence: NP_001132940.1). All available first- and second-degree relatives of the probands were tested for mutations (Figure 1) and assessed clinically (Table 1).

**Table 1.** Characteristics of the Probands and Relatives With a *TBL1X* Mutation Identified by Sanger Sequencing

Case	Sex	Nucleotide Alteration	Amino Acid Alteration	Age at Diagnosis of CeH, y	Age at Confirmation *TBL1X* Mutation, y	TSH, mU/L, Without Treatment (RI, 0.5–5.0)	FT4, pmol/L, Without Treatment (RI, 10–23)	BMI	Thyroid Volume on Ultrasound (RI)
A.II.3	M	c.1246A>T	N365Y	—	53	1.50	10.2	21.4 kg/m^2^	8.1 mL (4.9–19.1)
A.II.6	F	c.1246A>T	N365Y	—	51	3.40	10.4	25.4 kg/m^2^	8.3 mL (4.9–19.1)
A.III.6	F	c.1246A>T	N365Y	27	29	2.50	8.7	25.5 kg/m^2^	4.4 mL (4.9–19.1)
A.III.8	M	c.1246A>T	N365Y	2 wk^a^	25	1.8	6.8^b^	24.3 kg/m^2^	3 mL (4.9–19.1)
A.IV.1	M	c.1246A>T	N365Y	2 wk^a^	1.5	4.0 (1.7–7.9)	6.7 (12–30)	−1.2 SDS	0 mL^c^ (p2.5: 1)
A.IV.2	F	c.1246A>T	N365Y	—	1.5	1.90	15.4	+0.4 SDS	1.8 mL (p50: 2)
B.II.4	F	c.1510C>T	H453Y	—	42	3.0	16.3	44.4 kg/m^2^	17.3 (4.9–19.1)
B.III.4	M	c.1510C>T	H453Y	2 wk^a^	2	6.8 (1.7–7.9)	11.2 (12–30)	+0.6 SDS	0.9 mL (p2.5: 1)
C.I.1	M	c.1249G>A	A366T	—	50	1.70	13.5	24.8 kg/m^2^	9.3 mL (4.9–19.1)
C.II.1	F	c.1249G>A	A366T	14	15	2.4	6.8	+2.1 SDS	4.9 mL (4.9–19.1)
C.II.2	F	c.1249G>A	A366T	—	13	2.7	11.9	+1.5 SDS	5.2 mL (p50: 7.4)
C.II.3	F	c.1249G>A	A366T	—	10	1.9	12.5	−0.1 SDS	3.9 mL (p50: 5.2)
D.II.2	F	c.1526A>G	Y458C	—	50	0.44	14.3	33.1 kg/m^2^	14.7 mL (4.9–19.1)
D.III.1	M	c.1526A>G	Y458C	6	16	1.2	7.8	+4.1 SDS	3.3 mL (4.9–19.1)
E.I.2	F	c.1312-1G>A	Splice	—	48	2.8	15.0	32.5 kg/m^2^	8.2 mL (4.9–19.1)
E.II.1	M	c.1312-1G>A	Splice	14	17	2.38	7.0^d^	+0.7 SDS	6.1 mL (4.9–19.1)
E.II.2	F	c.1312-1G>A	Splice	15	15	3.20	9.5	+2.0 SDS	7.8 mL (p50: 9.1)
F.I.2	F	c.1258T>C	W369R	—	22	1.40	13.4	21.9 kg/m^2^	6.3 mL (4.9–19.1)
F.II.1	M	c.1258T>C	W369R	2 wk^a^	6 mo	2.30 (1.7–7.9)	7.9 (12–30)	+0.8 SDS	x

Abbreviations: F, female; L, left; M, male; R, right; RI, reference interval; x, missing value. Dashes indicate central hypothyroidism is not present. Reference intervals for FT4 are 10–23 pmol/L and in neonates, 12–30 pmol/L. Reference intervals for TSH are 0.5–5 mU/L and in neonates, 1.7–7.9 mU/L. BMI is expressed as kilograms per square meter or SDS calculated with Dutch reference data (20). Reference intervals for thyroid size for age are reported elsewhere (22).

a Detected by neonatal screening.

b Determined at the age of 3 months.

c Thyroid tissue too small to measure reliably.

d Measurements done in laboratory with different reference values.

The variant identified in family A (Figure 1A) was also found in three relatives. A second mutation was found in a 2.5-year-old boy with CeH (Figure 1B; B.III.4), detected through the Dutch neonatal CH screening, and in his mother. A third mutation was found in a 15-year-old girl (Figure 1C; C.II.1) diagnosed with CeH because of fatigue, weight gain, and secondary amenorrhea. The mutation was also found in her father and two sisters. The fourth mutation was found in a 16-year-old boy (Figure 1D; D.III.1), diagnosed with CeH after presenting with obesity, concentration difficulties, and macrocephaly. His mother had the same mutation. The fifth mutation was found in a 17-year-old boy (Figure 1E; E.II.1) diagnosed with CeH after presenting with short stature. The mutation was also found in his mother and sister. The sixth mutation was found in a 6-month-old boy (Figure 1F; F.II.1), detected through the Dutch neonatal CH screening, and in his mother. X-inactivation analysis showed absence of skewing in A.III.6, C.II.1, and E.II.2 (diagnosed with CeH) and A.II.6, B.II.4, and C.II.2 (low normal FT4 concentrations).

Sanger sequencing failed to show a variant in any of the genes known to cause CeH. Using WES, the three affected members of family A shared variants in eight genes (Supplemental Table 2A). Mutations in four genes are not known to be associated with a specific phenotype. The phenotypes associated with mutations in the other four genes were not present in our patients and are likely not causative. WES in the six patients of families A–D yielded variants in six genes, two of which cause phenotypes with dominant inheritance not present in our patients (Supplemental Table 2B). Three of the remaining four genes (*BRD8, NCOA6, MED15*) may play a role in mediating TH-dependent activation of gene transcription (16–18). Sanger sequencing showed that two of the five relatives with a TBL1X mutation and a low normal FT4 had the same variant as the proband in their family, and three did not (Supplemental Table 2B). There was no relation between FT4 concentrations and the presence or absence of variants, suggesting the absence of synergy of *TBL1X* mutations and variants. Mutations in the fourth gene (*THRB*) have been associated with resistance to thyroid hormone, accompanied by elevated TH levels. This was not present in our patients.

### Clinical phenotyping

#### Endocrine and anthropometric findings

**Figure 3.**
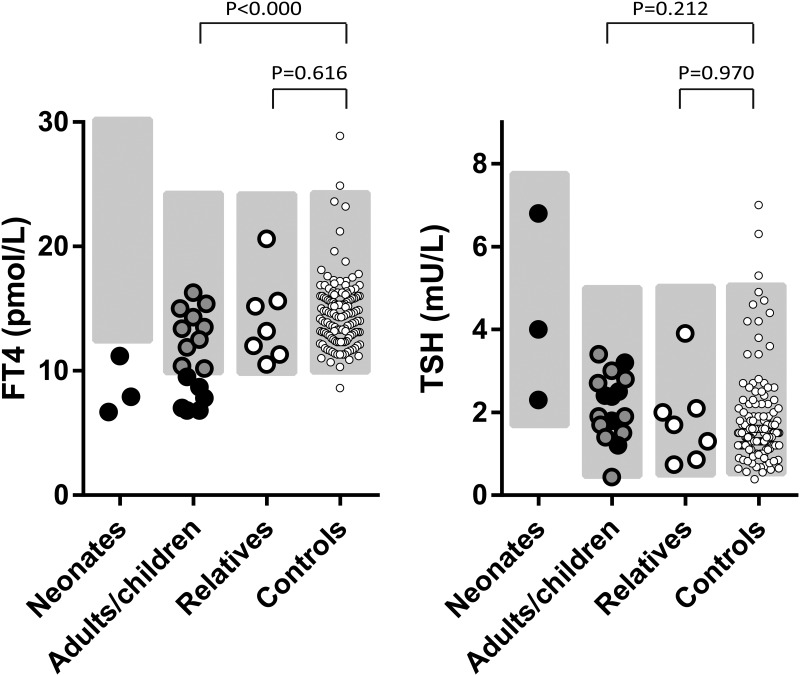
Graphic representation of plasma FT4 and TSH concentrations. Plasma FT4 (left panel) and TSH concentrations (right panel) in untreated condition are shown. Neonates are those with a *TBL1X* mutation. Adults/children are adults and children with a *TBL1X* mutation (black filled symbols: diagnosed with CeH; gray filled symbols: biochemically euthyroid). Relatives are adult relatives without a *TBL1X* mutation. Controls are adult controls. The gray-shaded areas denote the reference intervals. Reference interval for FT4 is 10–23 pmol/L; in neonates, 12–30 pmol/L; for TSH, 0.5–5 mU/L; in neonates 1.7–7.9 mU/L. The adult controls (n = 136) were recruited for earlier studies for the express purpose of establishing reference intervals. They were all healthy subjects, not suspected of an endocrine disorder.

The six probands and two members from family A had previously shown biochemical evidence of CeH, with FT4 concentrations between 56% and 93% of the lower limit of the reference interval (Table 1 and Figure 3). All were treated with LT4 during phenotyping. Ten individuals with a mutation (two males) had FT4 concentrations within the lower half of the reference interval (Tables 1 and Figure 3). E.II.2 had a FT4 concentration below the lower limit of the reference interval, without complaints. She is currently being monitored. Compared with a large adult control group, the FT4 concentrations of the 16 adults with a mutation were clearly and significantly lower (same FT4 assay, same laboratory; 11.2 vs 14.6 pmol/L, *P* < .000) (Figure 3). The seven relatives without a mutation had FT4 concentrations within the reference interval, similar to the controls (14.1 vs 14.6 pmol/L; *P* = .616) (Supplemental Table 3 and Figure 3). T_3_ concentrations were normal in all individuals with a mutation (Supplemental Table 4). Thyrotropin-releasing hormone (TRH) stimulation testing before LT4 treatment performed in six individuals with a mutation and CeH showed normal timing and peak concentration of TSH (Supplemental Table 5) (19), indicating intact responsiveness of the pituitary gland to TRH.

All individuals with a mutation had a normal height (20) and normal age at onset and progression of puberty. Body mass index (BMI) was greater than 30 kg/m^2^ in three of the nine adults and +2 or greater SD score (SDS) in three of the 10 children (20). Biochemical evaluation of the endocrine axes other than the HPT axis was normal (Supplemental Table 4).

### Imaging

Brain magnetic resonance imaging performed in six individuals with a mutation and CeH showed normal hypothalamic and pituitary morphology. Adolescent and adult males had a normal testicular size (21). Thyroid ultrasonography of seven of eight individuals with a mutation who were treated with T_4_ showed thyroid volumes below the percentile of 2.5 of age-specific reference intervals. Nine of 11 individuals with a mutation who were not treated with LT4 had thyroid volumes below the mean of age-specific reference intervals (22).

### Audiometry

**Figure 4.**
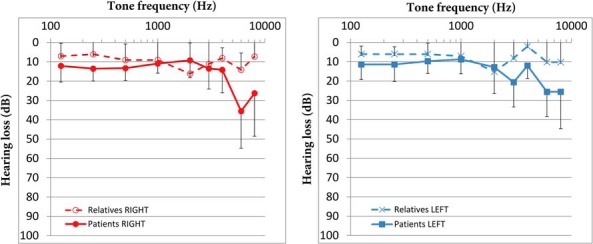
Mean and SD of decibels of hearing loss. Hearing loss per frequency in the right (left panel) and left ear (right panel) of individuals with a mutation (solid line) and relatives without a mutation (dashed line).

Individuals with a mutation had poorer hearing thresholds at high frequencies in PTA than their relatives without a mutation (Figure 4 and Supplemental Table 6), but the difference did not reach statistical significance. In total, 11 of the 15 individuals with mutations evaluated with PTA had hearing thresholds poorer than the age-specific reference interval (23). There was no correlation between the severity of the hearing loss and FT4 concentrations.

Of the three children evaluated with otoacoustic emission testing, one proband had poorer hearing thresholds relative to reference ranges, whereas two relatives with a mutation (one with CeH) had hearing thresholds at the lower limit of normal (24). Transiently evoked otoacoustic emissions and distortion product otoacoustic emissions were analyzed in 6-month-old F.II.1, which showed no abnormalities. The main clinical manifestation of hearing loss was having difficulties in following a conversation in a noisy environment.

### Liver enzymes and lipids

Liver enzymes were normal in all individuals with mutations except D.III.1, who showed slight elevation most likely caused by obesity (Supplemental Table 7). Low-density lipoprotein or total cholesterol concentrations were above the upper limit of age-specific reference intervals in one of the eight evaluated individuals with a mutation and CeH and in five of the nine evaluated individuals with a mutation without CeH (Supplemental Table 7). Because family C had a positive family history of early cardiovascular disease, C.II.2 was screened for familial hypercholesterolemia, which did not demonstrate mutations in the *LDLR* and the *APOB* genes.

### Protein structural analyses

**Figure 5.**
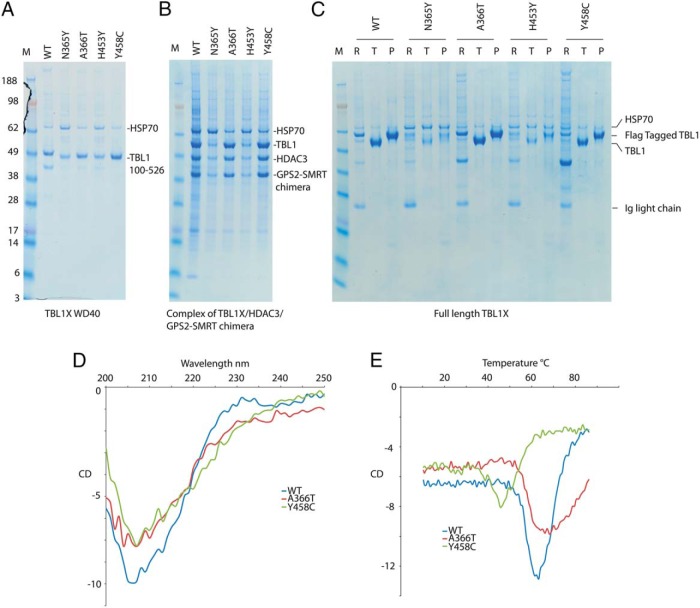
Transient expression in HEK293 cells and small-scale purification of wild-type and mutated TBL1X. A, SDS-PAGE of the purification of the isolated wild-type TBL1X and mutant WD40 domains. B, SDS-PAGE of the purification of the wild-type and mutant TBL1X-HDAC3-GPS2-SMRT chimera complex. C, Transient expression in HEK293 cells and small-scale purification of wild-type and mutated full-length TBL1X. SDS-PAGE of the purification of the TBL1X, R is the resin, T is the TEV-eluted protein, and P is the peptide-eluted protein. D, CD spectra from 250 to 200 nm at 20°C of wild-type, A366T, and Y458C TBL1X WD40 domains. E, Melting curves at 215 nm from 10°C to 90°C of wild-type, A366T, and Y458C TBL1X WD40 domains.

To investigate the functional consequences of the mutations in families A–D in the TBL1X WD40 domains, we expressed isolated WD40 domains and the full-length proteins. We also coexpressed full-length proteins with interaction partners GPS2/SMRT and HDAC3 (Figure 5).

TBL1X proteins containing the mutation N365Y or H453Y were poorly expressed compared with wild-type protein and were associated with elevated expression of the chaperone heat shock protein 70 kDa, suggesting that these mutations result in aberrant protein folding or stability. This fits well with their largely buried location in the structure of the WD40 domain, such that mutation to a larger tyrosine side chain cannot be tolerated. Due to the poor expression of the N365Y and H453Y proteins, we were unable to further analyze their structure and functional characteristics. We note that the entirely buried location of W369 would strongly suggest that mutation of this residue to an arginine would have a strongly deleterious effect on protein folding and stability.

In contrast, in all three contexts, wild-type TBL1X and TBL1X bearing the A366T and Y458C mutations were readily expressed and purified, either in isolation or in complex with partner proteins.

To investigate whether the A366T and Y458C mutations result in an altered protein structure or stability, we performed circular dichroism (CD)-monitored thermal denaturation studies of the WD40 domains (Figure 5). The wild-type and A366T and Y458C mutant WD40 domains showed CD spectra characteristic of their largely β-sheet structure. Both wild-type and A366T mutant WD40 domains underwent a cooperative denaturation at 70°C. Together these data suggest that this protein is correctly folded. In contrast, the Y458C mutation appeared to undergo thermal denaturation at a lower temperature, suggesting reduced thermal stability that may in part be responsible for its impaired biological function. We also performed a proteomic analysis of proteins that copurified with the A366T and Y458C mutant proteins and compared this with wild-type protein. In all cases the proteins associated with endogenous proteins known to be part of the corepressor complex. The consistent difference between the A366T and Y458C mutants and wild-type proteins was that the mutants showed association with cytoplasmic cytoskeletal proteins, suggesting that the relative nuclear vs cytoplasmic localization may be perturbed.

### RT-PCR, Western blotting, and immunocytochemistry

**Figure 6.**
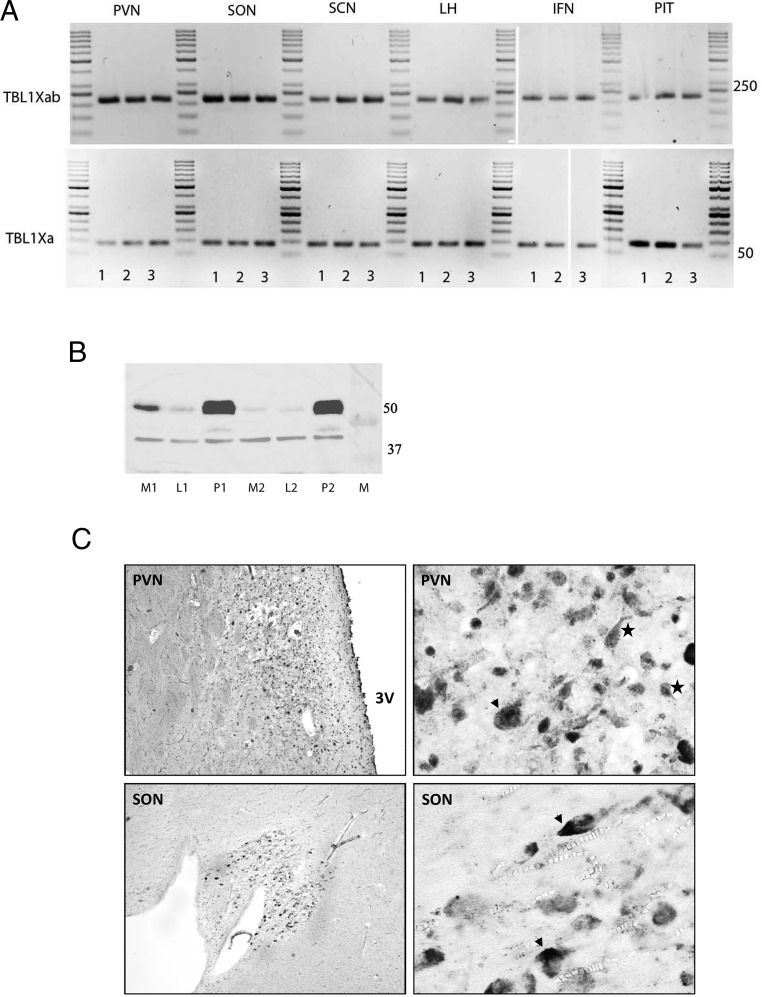
Expression of TBL1X in human brain tissue. A, TBL1X variants TBL1Xa and TBL1Xab transcript PCR product of three subjects (1, 2, and 3) on 2% agarose gel. The expected products are 83 bp (TBL1Xa) and 216 bp (TBL1Xab). INF, infundibular nucleus; LH, lateral hypothalamus; PIT, pituitary; SCN, suprachiasmatic nucleus; SON, supraoptic nucleus. B, Western blot of TBL1X (55 kDa) in the median hypothalamus (M), lateral hypothalamus (L), and pituitary (P) of two subjects (1 = 97–90 and 2 = 97–235; see Supplemental Table 8 for further details). β-Actin is used as a housekeeping protein (37 kDa), and the blot contains 10 μg protein of each sample. C, Representative TBL1X immunostaining in the hypothalamus of subject 2013-083. Arrowheads, Magnocellular neurons; stars, parvocellular neurons. SON, supraoptic nucleus; 3V, third ventricle. In the left upper panel, the PVN is visible just lateral to the third ventricle. The higher magnification shows darkly stained, mostly parvocellular and magnocellular neurons. The left lower panel shows an overview of the SON, and the higher magnification in the right lower panel shows darkly staining, mostly magnocellular neurons.

TBL1X mRNA expression was present in the pituitary and in hypothalamic nuclei (suprachiasmatic nucleus, supraoptic nucleus, paraventricular nucleus, infundibular nucleus, and lateral hypothalamic area) of each hypothalamus studied (Figure 6A). A Western blot showed clear bands in the pituitary (n = 2) and hypothalamus (n = 2) at the expected height (55 kDa) for TBL1X (Figure 6B). TBL1X immunostaining was present throughout the hypothalamic gray. In particular, prominent neuronal staining was present in the paraventricular and supraoptic nucleus (Figure 6C).

## Discussion

In this study we identified six missense mutations in *TBL1X* in eight patients (six males) from six families previously diagnosed with CeH and in 11 of their relatives (two males). Only one of these relatives met the biochemical criteria of CeH (E.II.2). The other 10 relatives had plasma FT4 concentrations in the lower half of the reference interval. As a group, the 16 adult mutation carriers had 20%–25% lower plasma FT4 concentrations than controls, whereas relatives without a mutation had FT4 concentrations similar to controls. All individuals with a mutation and CeH had a very small thyroid gland, similar to patients with *TSHB* and *IGSF1* mutations (25, 26). In combination with these very small thyroid glands and relatively low thyroid volumes in individuals with a mutation without CeH, the lowered FT4 concentrations are suggestive of longstanding, lower-than-normal TSH stimulation. *TBL1X* has not been associated with CeH or lower-than-average FT4 concentrations before. In addition, 12 of 19 evaluated individuals with a mutation had mild to profound hearing loss.

TBL1X consists of an N-terminal tetramerization domain and a WD40 domain. Whereas the tetramerization domain mediates assembly of the NCoR-SMRT corepressor complex (27), the WD40 domain is thought to be involved in mediating interaction of the complex with chromatin, enabling efficient histone deacetylation (28, 29). All mutations are in the WD40-repeat domains of TBL1X. Our protein studies suggest that the N365Y and H453Y mutations impair the folding or stability of the WD40 domain. Although the A366T and Y458C behaved very similar to the wild-type protein, the mutant's association with cytoplasmic cytoskeletal proteins suggests that the relative nuclear vs cytoplasmic localization may be perturbed. We speculate that the A366T and Y458C mutations impair interaction with partner proteins or chromatin. Taken together, these studies suggest that these four mutations alter the structural and functional properties of TBL1X.

We found *TBL1X* mRNA and TBL1X protein expression in the hypothalamic paraventricular nucleus (PVN) and pituitary gland. The prominent expression of TBL1X in the parvocellular neurons of the PVN suggests coexpression with TRH (30).

TBL1X is an essential subunit of the NCoR-SMRT complex, the major TR CoR involved in T_3_-regulated gene expression. This complex mediates the ability of the TR to repress the transcription of positively regulated T_3_ target genes in the absence of T_3_ (6). CoRs are additionally known to enhance TR-mediated basal activation of negatively regulated genes (such as *TRH* and *TSHB*) in the absence of T_3_, although the exact mechanism is only partly understood (7). In mice expressing a mutated NCoR protein resulting in a defective NCoR-SMRT complex, serum TH concentrations were decreased by 30%, whereas TSH was normal (6). This implies that the NCoR-SMRT complex is essential for adequate HPT axis regulation. Similar defective NCoR/SMRT complex functioning may very well be the underlying mechanism of the lowered FT4 concentrations in individuals with a mutation. We propose that a defective NCoR-SMRT complex is less able to activate transcription of negatively regulated genes in the absence of T_3_, resulting in decreased *TRH* and *TSHB* transcription, ultimately leading to decreased TH synthesis.

Because not all individuals with a mutation had CeH, one may hypothesize that mutations in genes other than *TBL1X* might be causally involved. We checked this by WES targeted for genes known or presumed to be involved in HPT axis sequencing (Supplemental Table 2C). In three of six patients with CeH and a mutation in *TBL1X,* WES demonstrated variants in such genes (Supplemental Table 2, A and B). However, these variants did not cosegregate with the CeH phenotype and were also present in individuals with a *TBL1X* mutation and a low normal FT4 concentration. This makes it unlikely that those variants are involved in the pathogenesis of the observed CeH. An alternative explanation for the wide range of FT4 concentrations of TBL1X mutation carriers may be that every healthy individual has his/her own optimal and stable FT4 concentration within the population reference interval (also known as the individual set point [31–33]), determined by both genetic and environmental factors. In the present series of patients, the *TBL1X* mutations result on average in a 3- to 4-pmol/L lower FT4 concentration. One may hypothesize that without a mutation in TBL1X, the CeH patients might not have had CeH but a FT4 concentration in the lower tertile of the population reference interval. Their relatives would have had values dispersed normally within this reference interval. Finally, although a skewed X-inactivation was not present in peripheral mononuclear cells, it may be present in other tissues. A varying expression of TBL1X in the hypothalamus may also have caused the observed variation in phenotype.

Although the normal TSH concentrations in the CeH patients may seem unexpected at first sight, this is commonly seen in CeH (3–5) and may be explained by altered glycosylation of the TSH protein, resulting in diminished bioactivity (1).

Hearing loss was previously reported in two unrelated patients with partial deletion of *TBL1X* (7, 8). However, the cause of the observed sensorineural hearing loss is unclear. Given the expression of *TBL1X* in mouse cochlea (7), a mutated TBL1X protein may have local detrimental effects on cochlear function or its development. Mutations in another subunit of the NCoR-SMRT complex, *TBL1XR1*, were found to cause hearing loss as well (34). Alternatively, because TH plays a crucial role in fetal inner ear maturation (35), the hearing loss may have resulted from the congenital hypothyroidism per se. However, the relatively mild hypothyroidism and lack of correlation of the severity of hearing loss with FT4 concentrations may argue against this.

In mice, loss of hepatic TBL1X was found to result in hepatic hypertriglyceridemia and steatosis (9). None of the present individuals with a mutation had hypertriglyceridemia or signs of hepatic steatosis. Because 6 of 17 had hypercholesterolemia, at this point we cannot exclude that hypercholesterolemia is part of the *TBL1X* mutation phenotype. In a genome-wide association study, *TBL1X* was identified as a candidate gene for male autism spectrum disorder (36), but none of present patients had been diagnosed with autism spectrum disorder.

An important question is whether the lowered plasma FT4 concentrations in individuals with *TBL1X* mutations, especially in those biochemically classified as having CeH, result in hypothyroidism at the level of TH target tissues. Earlier studies in mice expressing mutated NCoR suggested increased sensitivity to TH in peripheral tissues (6), and it is tempting to speculate that the same mechanism is present in individuals with a *TBL1X* mutation. This might also explain why patients who were diagnosed at a later age developed well intellectually and reached normal adult heights. Another intriguing question is whether the defective NCoR-SMRT corepressor complex function has consequences for the intrinsic action of other nuclear receptors, such as retinoic acid receptor and retinoid X receptor.

In conclusion, we demonstrate that mutations in *TBL1X* are associated with CeH and hearing loss. At this point it remains unclear whether these patients display hypothyroidism at the tissue level. Further studies are clearly needed to address this issue.
